# Resposta Vascular da Triiodotironina sobre Anéis de Aortas Isoladas: Contribuição de Mecanismos Redox

**DOI:** 10.36660/abc.20230236

**Published:** 2024-04-11

**Authors:** Viviane Cristina Pederiva, Alexandre de Castro, Adriane Belló-Klein, Alex Sander da Rosa Araujo, Patrick Turck

**Affiliations:** 1 Universidade Federal do Rio Grande do Sul Departamento de Fisiologia Laboratório de Fisiologia Cardiovascular Porto Alegre RS Brasil Universidade Federal do Rio Grande do Sul - Departamento de Fisiologia, Laboratório de Fisiologia Cardiovascular, Porto Alegre, RS – Brasil

**Keywords:** Tri-Iodotironina, Hormônios Tireóideos, Aorta, Homeostase

## Abstract

**Fundamento:**

A disfunção vascular constitui a etiologia de diversas doenças, incluindo infarto do miocárdio e hipertensão, diante da ruptura da homeostase oxi-redutiva (“redox”), desempenhando um papel no desequilíbrio do mecanismo de controle vasomotor. Nosso grupo demonstrou anteriormente que os hormônios tireoidianos melhoram a sinalização da angiogênese, exercendo efeitos protetores sobre o tecido aórtico de ratos infartados.

**Objetivos:**

Investigar o papel da triiodotironina (T3) na resposta vascular, explorando seus efeitos em aortas isoladas e a presença de mecanismos redox vasculares.

**Métodos:**

Anéis aórticos isolados (endotélio intacto e desnudado) pré-contraídos com fenilefrina foram incubados com T3 (10^-8^, 10^-7^, 10^-6^, 10^-5^ e 10^-4^ M) e a tensão foi registrada usando um transdutor de deslocamento de força acoplado a um sistema de coleta. Para avaliar o envolvimento do estresse oxidativo, os anéis aórticos foram pré-incubados com T3 e posteriormente submetidos a um sistema de geração de espécies reativas de oxigênio (ROS) *in vitro*. O nível de significância adotado na análise estatística foi de 5%.

**Resultados:**

A T3 (10^-4^ M) promoveu o vasorrelaxamento dos anéis aórticos pré-contraídos com fenilefrina em endotélio intacto e desnudado. Os anéis aórticos pré-incubados na presença de T3 (10^-4^ M) também mostraram diminuição da vasoconstrição provocada pela fenilefrina (1 µM) em preparações de endotélio intacto. Além disso, o efeito vasorrelaxante da T3 (10-4 M) persistiu em anéis aórticos pré-incubados com éster metílico de N^G^-nitro-L-arginina (L-NAME, 10 µM), um inibidor inespecífico da NO sintase (NOS). Por fim, a T3 (10^-4^ M) exibiu, *in vitro*, um papel antioxidante ao reduzir a atividade da NADPH oxidase e aumentar a atividade da SOD nos homogenatos aórticos.

**Conclusão:**

A T3 exerce efeitos dependentes e independentes de endotélio, o que pode estar relacionado ao seu papel na manutenção da homeostase redox.

## Graphical abstract

**Figura Central:**
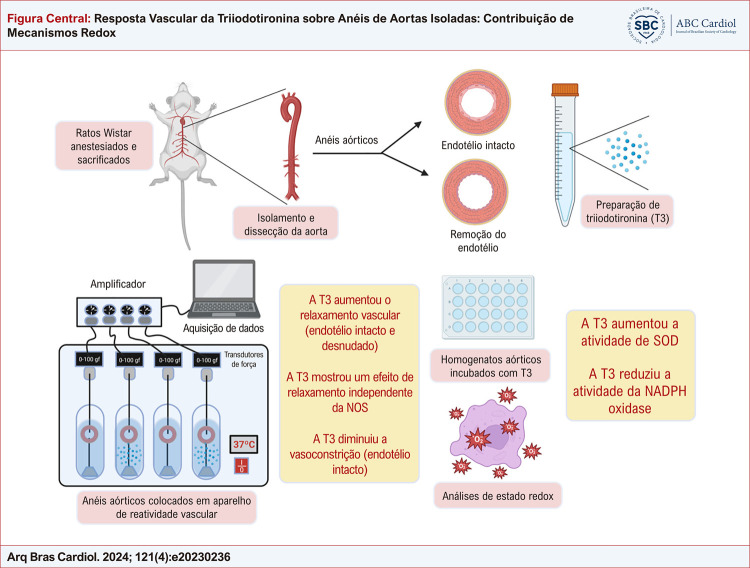
: Resposta Vascular da Triiodotironina sobre Anéis de Aortas Isoladas: Contribuição de Mecanismos Redox

A triiodotironina exerce um relaxamento vascular endotelial dependente e independente e diminui a vasocontração de anéis aórticos isolados, melhorando a homeostase redox vascular.

## Introdução

Em doenças cardíacas e cerebrais, alterações significativas são observadas nos mecanismos de relaxamento vascular, resultando na diminuição do fluxo sanguíneo, a qual é frequentemente associada a eventos isquêmicos.^1^ Condições como hipertensão arterial sistêmica, infarto do miocárdio, hipertensão pulmonar e acidente vascular cerebral são alguns dos exemplos de lesões vasculares vinculadas à vasoconstrição e comprometimento do sistema circulatório.^2^ A resposta exacerbada às catecolaminas, mediadores inflamatórios e espécies reativas de oxigênio (ROS) desempenham um papel crucial na disfunção endotelial.^3^ Nesse contexto, o estresse oxidativo assume uma importância significativa, visto que as ROS induzem vasoconstrição e efeitos adversos no remodelamento vascular.^4^ Adicionalmente, o aumento do tônus vascular gera maior resistência ao fluxo sanguíneo, resultando em tensão de cisalhamento. Esta condição exerce efeitos críticos sobre a homeostase endotelial, causando produção elevada de ROS. Diversas fontes celulares podem promover a formação de ROS, como mitocôndrias, retículo endoplasmático e peroxissomos. Além disso, enzimas como a xantina oxidase e a nicotinamida adenina dinucleotídeo fosfato oxidase (NADPH oxidase/Nox) também têm a capacidade de sintetizar essas espécies químicas.^5^

A NADPH oxidase é a principal enzima sintetizadora do radical ânion superóxido (O_2_^•−^) nos vasos.^6^ A molécula de O_2_^•−^ pode ser convertida em peróxido de hidrogênio (H_2_O_2_) em uma reação catalisada pela superóxido dismutase (SOD). A partir dessa perspectiva, o H_2_O_2_ desempenha um papel duplo, sendo capaz de não somente produzir vasodilatação nas artérias cerebrais, coronárias e pulmonares,^7^ como também de atuar como vasoconstritor relevante nas artérias periféricas e na aorta.^8^ Além disso, o O_2_^•−^ combina-se ao óxido nítrico (NO), resultando na formação de peroxinitrito (ONOO^−^).^9^ O ONOO^−^ é um potente agente oxidante de membranas biológicas, capaz de induzir a morte celular. Assim, encontrar abordagens terapêuticas que combinem ações vasodilatadoras e antioxidantes é importante para o tratamento de doenças vasculares.^10^

Os hormônios tireoidianos (TH) demonstraram promover efeitos cardioprotetores e vasoprotetores em modelos experimentais de doenças cardíacas. No modelo de infarto, o TH não apenas melhorou o remodelamento cardíaco adverso pós-infarto, como também apresentou ação antioxidante sobre o tecido cardíaco e vascular^11,12^ além de melhorar a expressão de proteínas de angiogênese, como o fator 1α induzível por hipóxia (HIF-1α) e o fator de crescimento endotelial vascular (VEGF).^12^ Além disso, a T3 induz o vasorrelaxamento por meio de diferentes mecanismos. Em um modelo experimental de dieta com sobrecarga de sal, os vasos incubados com T3 aumentaram sua vasodilatação e diminuíram a produção de ROS.^13^ Elevação dos níveis de NO e a ativação das vias da proteína quinase G (PKG)/fosfoproteína estimulada por vasodilatador (VASP) e fosfoinositídeo-3-quinase (PI3K)/ proteína quinase B (AKT) parecem ser o principal processo pelo qual o TH promove o vasorrelaxamento.^14^ No entanto, a T3 também exerce sua função vasodilatadora de maneira independente do endotélio, sugerindo uma ação direta do TH sobre as células musculares lisas.^15^

Apesar de estudos demonstrarem ação protetora do TH no coração e nos vasos,^11,12^ ainda se faz necessário elucidar o papel da T3 sobre o tônus vascular e sobre o estresse oxidativo nos vasos. Assim, é necessário explorar ainda mais o mecanismo vasodilatador da T3 e o impacto das ROS neste processo. Para tanto, o objetivo deste estudo foi avaliar o impacto da T3 sobre a resposta vascular em vasos isolados quanto à influência do endotélio e o envolvimento dos parâmetros de estresse oxidativo neste contexto.

## Métodos

### Animais

Foram utilizados ratos Wistar machos com 45 dias de idade (pesando 202 ± 25 g) (N = 60), provenientes do Centro de Reprodução e Experimentação de Animais de Laboratório. Os animais foram alocados em condições padrão de biotério: ambiente com temperatura controlada (21 ± 2 °C), ciclo claro-escuro de 12 horas e umidade relativa de 70%; ofereceu-se água e ração padrão para roedores “ad libitum”. Os animais também foram aclimatados durante sete dias antes do início do protocolo experimental. Todos os procedimentos deste estudo estavam de acordo com as diretrizes para Pesquisa Biomédica Envolvendo Animais do Conselho de Organizações Internacionais de Ciência Médica (CIOMS) e foram aprovados pelo Comitê de Ética no Uso de Animais (número do protocolo: 38964).

### Cálculo do tamanho da amostra

O cálculo do tamanho da amostra foi realizado utilizando o software G*Power 3.1.9.2 (Schleswig-Holstein, Alemanha). Foram consideradas probabilidade de erro α = 0,05, poder de teste estatístico = 0,80 e tamanho do efeito = 0,4.

### Preparação dos vasos para a reatividade vascular

Os ratos foram anestesiados com cetamina (90 mg/kg, intraperitoneal) e xilazina (10 mg/kg, intraperitoneal) simultaneamente e sacrificados por decapitação. Após a eutanásia, as aortas foram rapidamente isoladas e mantidas em solução tampão Krebs-Henseleit (NaCl 115 mM, CaCl_2_ 2,5 mM, KCl 4,6 mM, KH_2_PO_4_ 1,2 mM, MgSO_4_. 7 H_2_O 1,2 mM, NaHCO_3_ 25 mM, dextrose 11,1 mM, Na_2_EDTA 3 mM), a 37 °C e pH 7,4, sob 95% O_2_–5% CO_2_. Os tecidos adiposo e conjuntivo foram cuidadosamente removidos e as aortas foram cortadas em segmentos cilíndricos de 4 mm de comprimento. Em alguns experimentos, o endotélio foi removido mecanicamente, esfregando-se suavemente a superfície interna dos segmentos da aorta com um fio de aço inoxidável. Dois ganchos de fio de aço inoxidável dobrados em um formato de L modificado foram utilizados para montar cada anel em câmaras de banho de órgãos (AVS Projetos, São Carlos, SP, Brasil). A porção curta e reta de cada gancho foi passada através do lúmen do anel. O gancho inferior foi preso à base da câmara de órgão preenchida com 10 mL de solução tampão Krebs-Henseleit e o gancho superior a um extensômetro. A tensão isométrica foi registrada por meio de um transdutor de deslocamento de força (TIM-100; AVS Projetos, São Carlos, SP, Brasil) conectado a um sistema de coleta (AQCAD 2.0.5; AVS Projetos, São Carlos, SP, Brasil). Os segmentos foram submetidos a uma tensão basal de 1,0 gf, ajustada a cada 15 minutos durante um período inicial de equilíbrio de 45 minutos.

A integridade do endotélio foi verificada pela incubação com acetilcolina (10 µM). Um relaxamento igual ou superior a 90% da contração máxima induzida pela fenilefrina (1 µM) foi considerado demonstrativo da integridade funcional do endotélio. Cada anel foi lavado sequencialmente com solução tampão de Krebs-Henseleit, reequilibrado por 45 minutos, e as tensões basais foram registradas.

### Estudos da reatividade vascular

O protocolo de incubação experimental é representado da seguinte forma: a) verificação de uma curva dose-resposta de T3 (10^-8^a 10^-4^ M) em segmentos aórticos com endotélio intacto e desnudado, pré-contraídos com fenilefrina 1µM; b) pré-exposição de vasos com endotélio intacto e desnudado a T3 (10^-4^ M), para mostrar a menor resposta contrátil induzida pela fenilefrina; c) verificação da T3 (10^-4M^) em segmentos aórticos com endotélio intacto pré-contraídos com fenilefrina 1µM e expostos a um inibidor inespecífico da NO sintase (NOS), éster metílico de NG-nitro-L-arginina (L-NAME; 10 µM) por 20 minutos. A T3 foi diluída em tampão de Krebs-Henseleit e as soluções foram preparadas diariamente, ao abrigo da luz. O grupo controle consistiu na adição dos mesmos volumes de solução tampão de Krebs-Henseleit, sem T3.

### Preparação da aorta para ensaios in vitro

As aortas foram homogeneizadas por 40 segundos (OMNI Tissue Homogeneizer, OMNI International, Kennesaw, GA, EUA) na presença de tampão de RIPA a 1:10 (5 ml/g de tecido) com fluoreto fenil-metil-sulfonil (PMSF) (100 mM) a 1 % (v/v). O homogenato foi centrifugado por 10 min a 8.000 x*g* em centrífuga refrigerada (4 °C) (Sorvall RC 5B – Rotor SM 24, Sorvall, Waltham, MA, EUA) e o sobrenadante foi coletado para análise subsequente.

### Sistema de geração de radical hidroxila

Para verificar o papel da T3 no equilíbrio redox, os sobrenadantes homogeneizados da aorta foram incubados com T3 (10^-4^ M) por 15 min a 37 °C. O grupo que não recebeu T3 foi incubado com água destilada no mesmo volume. Em seguida, as amostras foram incubadas por 30 min a 37 °C com FeCl_2_ (20 µM), H_2_O_2_ (50 mM) e ácido ascórbico (1 mM) para induzir estresse oxidativo (sistema de geração de radical hidroxila).^16^ Essas amostras foram utilizadas para avaliação bioquímica

### Análises bioquímicas

#### Dosagem de proteínas

As proteínas foram quantificadas pelo método descrito por Lowry et al.^17^ e medidas em espectrofotômetro (Anthos Zenyth 200 rt, Biochrom, Cambridge, Reino Unido) a 625 nm. Os resultados foram expressos em mg/mL.

#### Determinação do total de ROS

Os níveis totais de ROS nos homogenatos de vasos foram detectados usando diacetato de 2’,7’-dicloro-dihidro-fluoresceína (DCFH-DA), conforme descrito anteriormente.^18^ O DCFH-DA foi oxidado em 2,7-diclorofluoresceína fluorescente (DCF) na presença de ROS. As amostras foram excitadas a 488 nm e as emissões foram coletadas com um filtro de 525 nm (Espectrômetro de Fluorescência LS 55, Perkin Elmer, Waltham, MA, EUA). Os resultados foram expressos em nmol DCF/mg de proteína.

#### Peroxidação lipídica

A peroxidação lipídica foi avaliada por meio da produção de quimiluminescência iniciada pela adição de hidróxido de terc-butila (TBOOH).^19^ A quimiluminescência foi medida em um espectrômetro de cintilação (LKB Wallac Rack Beta Liquid Scintilla Spectrometer 1215, Austrália) operando como luminômetro e com fototubo sensor de emissão na faixa de 380–620 nm. Os resultados foram expressos como contagens por segundo cps/mg de proteína.

#### Atividade da NADPH Oxidase

A atividade da NADPH oxidase foi baseada no monitoramento do consumo de NADPH a 340 nm (Anthos Zenyth 200 rt, Biochrom, Cambridge, Reino Unido). Os resultados foram expressos como nmol NADPH/min/mg de proteína.^20^

#### Atividade da superóxido dismutase (SOD)

A determinação da atividade da SOD foi baseada na inibição da reação do ânion radical superóxido com o pirogalol.^21^ Os resultados foram expressos como U SOD/mg de proteína.

#### Atividade da catalase

A atividade da catalase foi avaliada por meio da medição espectrofotométrica da taxa de decomposição do peróxido de hidrogênio a 240 nm.^22^ Os resultados foram expressos em nmol/min/mg de proteína.

## Análise estatística

Todos os conjuntos de dados foram submetidos ao teste de normalidade de Shapiro-Wilk. Experimentos de diferentes doses de T3 ao longo do tempo, em comparação com o grupo Controle, foram analisados por ANOVA fatorial, complementada com teste post-hoc de Bonferroni para comparações múltiplas. Experimentos comparando o efeito de pré-incubação de dose fixa de T3 (10^-4^ M) sobre a vasoconstrição provocada pela fenilefrina e o efeito dA T3 (10^-4^ M) em anéis aórticos pré-incubados com L-NAME, em comparação com o grupo Controle, foram analisados por testes T não pareados. Experimentos do equilíbrio redox foram analisados por meio do método ANOVA bidirecional e complementados com o teste post-hoc de Bonferroni para comparações múltiplas além dos níveis totais de ROS, que, por sua vez, foram analisados por meio do teste de Kruskal-Walli, complementado com o teste post-hoc de Dunn para comparações múltiplas. Os resultados foram expressos como média ± desvio padrão para todos os conjuntos de dados, exceto os níveis totais de ROS, cujos resultados foram plotados como um gráfico de pontos de dispersão, representando a mediana e o intervalo interquartil para cada conjunto de dados. O software GraphPad Prism 8.0.2 para Windows (GraphPad Software, San Diego, CA, EUA) foi utilizado para análise de dados. Os dados foram considerados significativos quando p < 0,05.

## Resultados

### A T3 promove relaxamento dos segmentos aórticos

**Figura 1:**
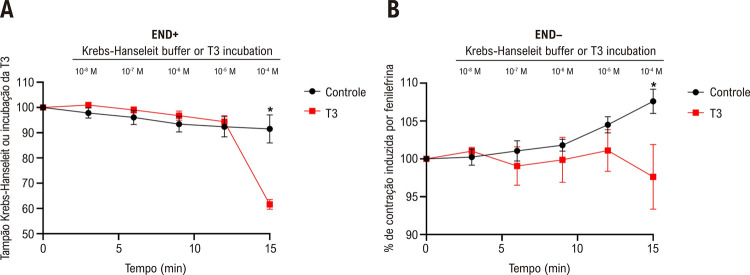
– Efeito da T3 sobre o relaxamento vascular dos segmentos aórticos (A) na presença de endotélio (END+) e (B) na ausência de endotélio (END-) pré-contraído com fenilefrina. Valores representados como média ± desvio padrão (DP), n = 6-8 anéis aórticos por grupo. ANOVA fatorial seguida de pós-teste de Bonferroni. *Diferença significativa entre os grupos Controle e T3 (10-4 M); p < 0,05.

Considerando o vaso intacto, a T3 a 10^-4^ M induziu o relaxamento das aortas pré-contraídas pela fenilefrina em comparação com o grupo controle (Figura 1A). A remoção do endotélio não anulou o efeito de vasorrelaxamento induzido pela T3 (10^-4^ M) (Figura 1B).

### A T3 diminui a resposta de vasocontração induzida pela fenilefrina

**Figura 2:**
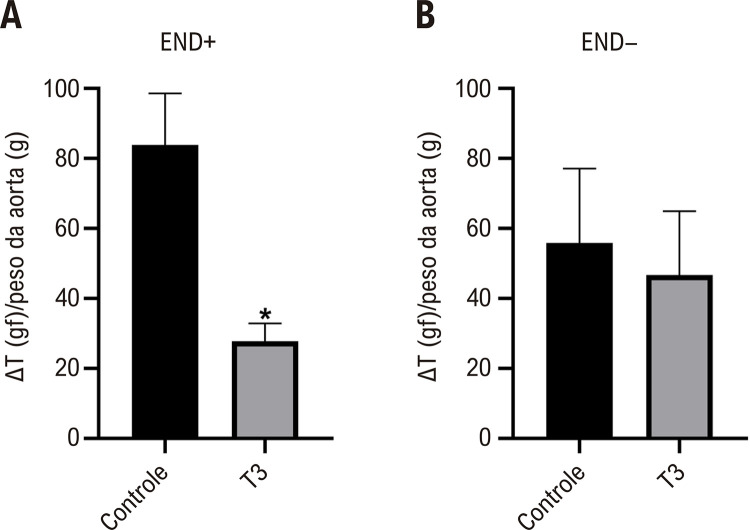
– Efeito da pré-incubação de T3 (10-4 M) sobre a vasocontração dos segmentos aórticos, provocada pela fenilefrina (A) na presença de endotélio (END+) e (B) na ausência de endotélio (END-). Valores representados como média ± desvio padrão (DP), n = 6-10 por grupo. Teste t não pareado. *Diferença significativa entre os grupos Controle e T3; p < 0,05. ∆T/peso da aorta = razão da variação da tensão pelo peso do vaso.

A pré-exposição dos segmentos aórticos intactos à T3 (10-4 M) resultou em uma redução da contração induzida pela fenilefrina em comparação com o grupo controle (Figura 2A). Porém, o mesmo resultado não foi observado nas preparações sem endotélio (Figura 2B).

### A T3 exibe um efeito vasorrelaxante independente de NOS

**Figura 3:**
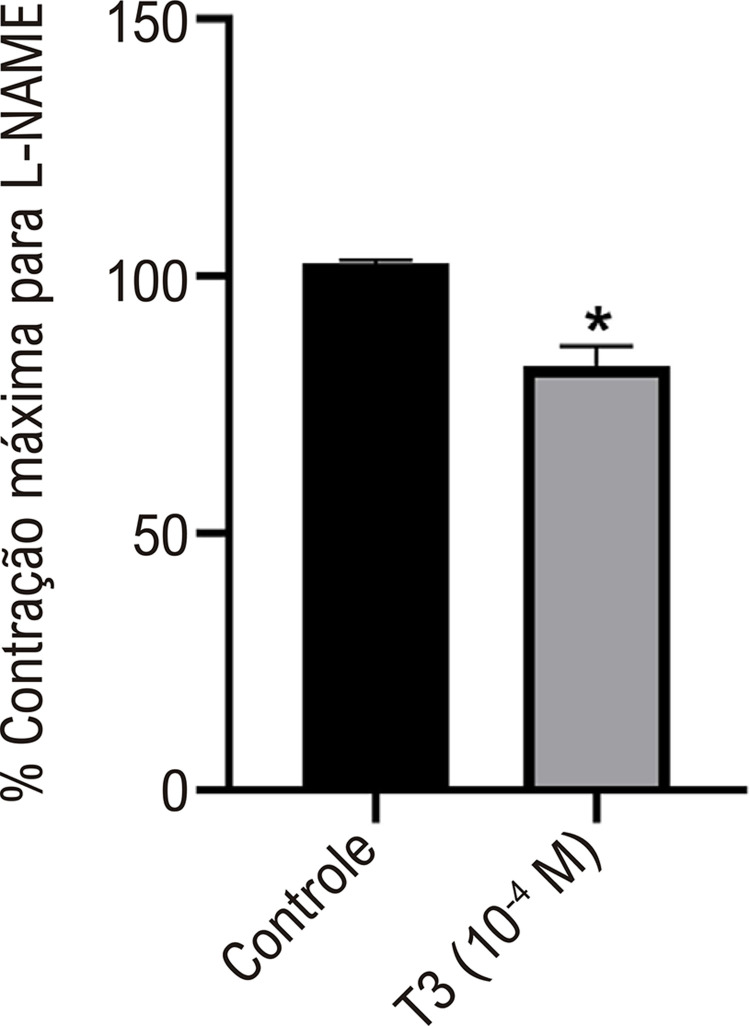
– Efeito da T3 (10-4 M) sobre o relaxamento vascular de anéis aórticos com endotélio intacto pré-incubados com L-NAME. Valores representados como média ± desvio padrão (DP), n = 6 por grupo. Teste t não pareado. *Diferença significativa entre os grupos Controle e T3; p < 0,05.

Vasos com endotélio intacto foram pré-incubados com L-NAME, um inibidor inespecífico de NOS, antes da incubação de T3 (10^-4^ M). Mesmo na presença de L-NAME, a T3 (10-4 M) ainda resultou em relaxamento (~20%) dos segmentos aórticos pré-contraídos induzidos por fenilefrina em comparação com o grupo controle (Figura 3).

### A T3 reduz a atividade da NADPH oxidase e melhora a atividade da SOD *in vitro*

**Figura 4:**
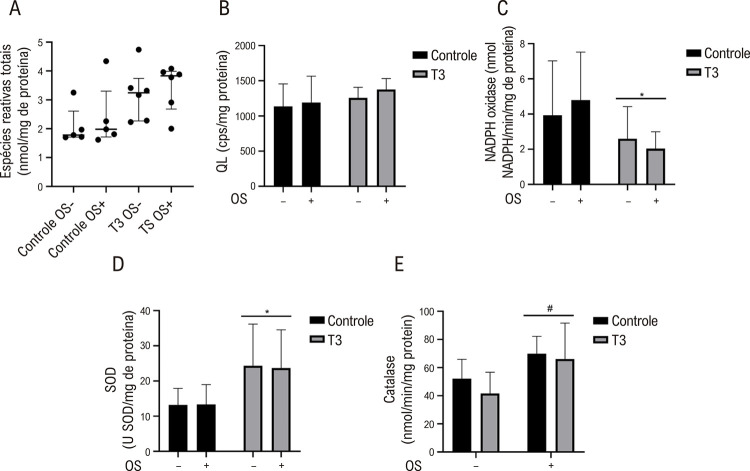
– Efeito da incubação de T3 (10-4 M) sobre (A) o nível de espécies reativas totais de oxigênio (ROS), (B) a oxidação lipídica, (C) a atividade da NADPH oxidase, (D) a atividade da superóxido dismutase (SOD) e (E) a atividade da catalase em homogenatos aórticos. Valores representados como mediana com intervalo interquartil para (A) e média ± desvio padrão (DP) para (B) a (E), n = 4-6 por grupo. Teste de Kruskal-Walli complementado com teste post-hoc de Dunn para (A) e ANOVA de dois fatores foi complementado com teste post-hoc de Bonferroni para (B) a (E). *Diferença significativa entre os grupos Controle e T3 na ANOVA de dois fatores; p < 0,05. #Diferença significativa entre os grupos (OS-) e (OS+); p < 0,05. OS- = grupos sem indução de estresse oxidativo (sistema de geração de radicais hidroxila); OS+ = grupos com indução de estresse oxidativo (sistema de geração de radicais hidroxila).

Embora a concentração total de ROS e a oxidação lipídica não difiram entre os grupos (Figura 4A e 4B), a atividade da NADPH oxidase foi reduzida quando as amostras foram expostas à T3 (10^-4^ M) em comparação com os grupos controle (Figura 4C). Observou-se um aumento da atividade da SOD em vasos incubados com T3 (10^-4^ M) em comparação com os grupos controle (Figura 4D). A atividade da catalase estava aumentada apenas nos grupos que receberam a indução do estresse oxidativo (Figura 4E).

## Discussão

Os principais resultados do presente estudo incluem a indução da vasodilatação de anéis aórticos com endotélio intacto e desnudado, pré-contraídos com fenilefrina em modelo experimental *ex vivo*, pela T3, cujo protocolo foi desenhado para avaliação da reatividade vascular. Além disso, a T3 desempenha um papel fundamental na homeostase redox, reduzindo a atividade da NADPH oxidase e aumentando a atividade da SOD dos vasos aórticos *in vitro* (Figura Central).

Estresse oxidativo, hipóxia, aterosclerose e inflamação são exemplos de desencadeadores de eventos vasoconstritores, os quais promovem desequilíbrio entre vasodilatação e vasoconstrição, remodelamento muscular vascular adverso e dano endotelial.^23^ Juntos, esses fatores promovem o aparecimento de diversas doenças como acidente vascular cerebral, infarto do miocárdio e hipertensão.^24^ Portanto, é importante explorar os mecanismos subjacentes à reatividade dos vasos de modo a propor alternativas terapêuticas que possam contribuir para o manejo das doenças vasculares. Nosso modelo experimental focou na avaliação da resposta vascular de aortas isoladas na presença de T3. A incubação com fenilefrina induziu a contração dos anéis aórticos, que foram posteriormente submetidos a diferentes doses de T3. Nossos resultados mostraram que a T3, na concentração de 10^-4^M, reduziu a vasoconstrição induzida pela fenilefrina. Corroborando com nossos dados, Carrillo-Sepulveda et al.,^13^ utilizando aortas de ratos Dahl (fêmeas) sensíveis ao sal, submetidos a uma dieta rica em sal, mostraram que a pré-incubação com T3 reduziu o estado hipercontrátil dos vasos e melhorou a resposta vasodilatadora. No modelo experimental de infarto do miocárdio, Ortiz et al.^12^ verificaram que o tratamento com T3 e T4 (2 e 8 μg/100 g/dia, respectivamente, por via oral por 12 dias) aumentou o imunoconteúdo de VEGF e HIF-1α (proteínas envolvidas na angiogênese em cenário hipóxico) na aorta de ratos infartados. Além disso, o VEGF também induz vasodilatação.^25^

Avaliando o envolvimento do endotélio sobre o efeito vasodilatador do TH, nossos resultados mostraram que a administração de T3 resultou em uma redução na constrição vascular induzida pela fenilefrina em aortas com e sem endotélio. Contudo, em aortas pré-incubadas com 10^-4^ M de T3, a ausência de endotélio afetou significativamente a capacidade de prevenir um aumento da tensão vascular evocada pela fenilefrina. Diante disso, é possível que, em vasos intactos, a T3 possa promover vasodilatação por meio de uma combinação de mecanismos dependentes e independentes do endotélio. Nos vasos sem endotélio, a vasodilatação é mantida por mecanismos independentes, mas a capacidade da T3 de reduzir a tensão vascular diminui. Estes resultados destacam a relevância do endotélio sobre o efeito total da T3 nos vasos. Devido a esse resultado, fez-se necessário investigar o papel do NO no processo de vasodilatação induzido pela T3, uma vez que o endotélio é uma importante fonte dessa molécula vasodilatadora.

O papel da T3 no endotélio e nos mecanismos vasodilatadores envolvidos nessa relação tem sido explorado nos últimos anos, destacando a relevância das vias de sinalização dependentes de NO.^26^ Além disso, o TH é capaz de aumentar a atividade da óxido nítrico sintase nos eritrócitos, favorecendo a produção de NO no sangue e melhorando a sua biodisponibilidade na circulação.^27^ No presente estudo, quando os anéis aórticos foram pré-incubados com L-NAME, um conhecido inibidor da NOS, a ação vasodilatadora da T3 persistiu. Esses dados corroboram a hipótese de que a T3 pode estar induzindo vasodilatação também por meio de um mecanismo independente do NO e do endotélio. Estes resultados não diminuíram a importância do endotélio sobre o efeito deste hormônio, eles demonstraram a capacidade da T3 em promover dilatação no contexto onde o endotélio é removido/danificado. Samuel et al.^28^ sugeriram que esse efeito independente do NO está associado à modulação da via da proteína quinase G/fosfoproteína, estimulada por vasodilatador (PKG/VASP) diretamente nas células musculares lisas vasculares. Além disso, o efeito antioxidante de baixas doses de hormônios tireoidianos também é capaz de melhorar a disfunção vascular e contribuir para um tônus vascular relaxado.^12,13^

Embora as ROS sejam importantes no controle do tônus vascular, quando suas concentrações excedem a capacidade antioxidante do tecido, o estresse oxidativo é estabelecido. A interrupção da homeostase redox resulta em danos vasculares, oxida lipoproteínas de baixa densidade e causa aterosclerose. O processo inflamatório associado a esses eventos leva ao remodelamento vascular adverso, à diminuição dos agentes vasodilatadores e ao aumento do nível de vasoconstrição.^29^ Nossos resultados mostram que a incubação com T3 reduziu a atividade do NADPH na aorta sob condições experimentais de estresse oxidativo estabelecidas *in vitro*. Além disso, a NADPH oxidase desempenha um papel importante na formação de ROS nos vasos sanguíneos. Essas espécies são capazes de promover um ambiente celular oxidado, levando ao desacoplamento da NOS e ao aumento da produção do radical ânion superóxido em vez do NO. Além disso, os radicais livres, como o ânion superóxido, muitas vezes atuam como sequestradores de NO, reduzindo sua biodisponibilidade e prejudicando a vasodilatação.^30^ Nesse sentido, Ortiz et al.^12^ também observaram uma redução na atividade da NADPH oxidase na aorta de ratos infartados tratados com hormônios tireoidianos, enquanto De Castro et al.^11^ mostraram que a administração de TH é capaz de reduzir ROS no coração de ratos infartados. Porém, em termos de níveis totais de ROS, o presente estudo não observou diferenças entre os grupos. Um resultado semelhante foi encontrado para avaliação da oxidação lipídica. Esses resultados podem ter surgido devido ao uso de aortas provenientes de animais saudáveis. Dentro deste contexto, essas amostras de vasos foram induzidas ao estresse oxidativo por meio da geração de modelo de hidroxila, no qual as amostras foram incubadas com FeCl_2_, H_2_O_2_ e ácido ascórbico por 30 min a 37°C. Entretanto, em estudo anterior avaliando a aorta de ratos infartados, a administração de TH promoveu diminuição dos níveis de ROS.^12^ Apesar disso, a capacidade da T3 de diminuir a atividade da NADPH oxidase já demonstra o efeito vasoprotetor desse hormônio. Além disso, quando avaliamos enzimas antioxidantes, a administração de T3 induziu aumento na atividade da SOD no tecido vascular. A SOD converte o ânion radical superóxido em peróxido de hidrogênio, reduzindo os níveis desse radical livre e sustentando um estado de aumento da biodisponibilidade de NO, melhorando assim a capacidade de vasorrelaxamento. Neste sentido, nossos resultados mostram que a T3 promove um cenário favorável à melhora da biodisponibilidade de NO, contribuindo para o mecanismo de vasodilatação dependente do endotélio. Esses dados corroboram outros estudos que mostram o papel vasoprotetor dos hormônios tireoidianos.^12^ Quando avaliamos a atividade da catalase, apenas as aortas submetidas ao estresse oxidativo apresentaram aumento na atividade dessa enzima. Estes resultados indicam um mecanismo compensatório do sistema antioxidante enzimático em resposta ao desequilíbrio redox; no entanto, a administração de T3 não modificou a atividade da catalase. Com relação a esse achado, um estudo investigando os efeitos dos TH em eritrócitos de ratos infartados revelou um aumento na atividade da catalase nas células sanguíneas dos animais tratados.^27^ Com base nisso, é plausível sugerir que, sob a influência da T3, os eritrócitos possam desempenhar um papel mais significativo na atividade dessa enzima antioxidante do que a própria parede vascular.

### Limitação do estudo

A impossibilidade de explorar a expressão proteica diretamente nos anéis aórticos utilizados na reatividade vascular foi uma limitação do estudo. A avaliação da expressão de proteínas, como a sintase do óxido nítrico endotelial (eNOS) e a via de sinalização PIK3/AKT, bem como de proteínas antioxidantes, como o fator 2 relacionado ao fator nuclear eritróide 2 (NRF2), na mesma amostra incubada com T3 e submetida aos protocolos de vasoconstrição e vasodilatação, teria contribuído para a compreensão da mecânica dos resultados observados. No entanto, o tamanho reduzido dos anéis aórticos e dos fios utilizados no protocolo de reatividade vascular reduzem o grau de confiabilidade para experimentos subsequentes de biologia molecular utilizando a mesma amostra. Outro aspecto limitante deste artigo é a falta de avaliação de outros vasos de resistência, como pequenas artérias e arteríolas, para verificar se o efeito da T3 na aorta poderia ser extrapolado. Na realidade, o estado contrátil das pequenas artérias e arteríolas é responsável pela definição da resistência vascular, sendo um determinante chave da pressão arterial. Estruturalmente, as artérias de resistência possuem uma ou duas camadas de células musculares lisas vasculares (CMLV), enquanto as artérias condutoras, como a aorta, possuem cerca de 15 camadas de CMLV. Nesse contexto, as células endoteliais das artérias de resistência projetam a membrana plasmática para a camada CMLV por meio da lâmina elástica interna (projeções mioendoteliais). As projeções mioendoteliais conectam-se às CMLV por meio de junções comunicantes que facilitam a comunicação elétrica entre as duas camadas celulares.^31^ Nesse contexto, a hiperpolarização das células endoteliais é transmitida às CMLV, relaxando os vasos; portanto, este é o principal mecanismo para a vasodilatação dependente do endotélio das artérias de resistência. Por outro lado, tal mecanismo difere para a aorta, por exemplo, uma vez que as projeções mioendoteliais muitas vezes estão ausentes nos vasos condutores. Assim, a vasodilatação mediada por NO é o mecanismo primário neste cenário.

## Conclusão

Com base nos dados obtidos, é possível concluir que a T3 exerce um importante papel vasodilatador, que se mantém mesmo na ausência do endotélio. Além disso, é capaz de diminuir a atividade da enzima pró-oxidante NADPH e aumentar a atividade da enzima antioxidante SOD. Estes resultados apontam para um efeito benéfico deste hormônio sobre o equilíbrio redox vascular e a resistência periférica. Esse cenário favorece maior biodisponibilidade de NO, contribuindo também para o mecanismo de vasodilatação dependente do endotélio. Dentro deste contexto, esses dados endossam outros estudos que demonstram efeitos benéficos dos hormônios tireoidianos sobre o sistema circulatório.
