# Supplementary material for: Resposta Vascular da Triiodotironina sobre Anéis de Aortas Isoladas: Contribuição de Mecanismos Redox
Source: Arq Bras Cardiol. 2024 Apr 11;121(4):e20230236. [Article in Portuguese] doi: 10.36660/abc.20230236 (PMC11098570; doi:10.36660/abc.20230236)
Supplement: Translation

# Translation: Vascular Response of Triiodothyronine on Isolated Aortic Rings: Contribution of Redox Mechanisms

**DOI:** 10.36660/abc.20230236i

**Authors:** Viviane Cristina Pederiva, Alexandre de Castro, Adriane Belló-Klein, Alex Sander da Rosa Araujo, Patrick Turck

**Affiliations:** 1 Brazil Universidade Federal do Rio Grande do Sul - Departamento de Fisiologia, Laboratório de Fisiologia Cardiovascular, Porto Alegre, RS – Brazil

**Keywords:** Triiodothyronine, Thyroid Hormones, Aorta, Homeostasis

## Abstract

**Background:**

Vascular dysfunction constitutes the etiology of many diseases, such as myocardial infarction and hypertension, with the disruption of redox homeostasis playing a role in the imbalance of the vasomotor control mechanism. Our group previously has shown that thyroid hormones exert protective effects on the aortic tissue of infarcted rats by improving angiogenesis signaling.

**Objective:**

Investigate the role of triiodothyronine (T3) on vascular response, exploring its effects on isolated aortas and whether there is an involvement of vascular redox mechanisms.

**Methods:**

Isolated aortic rings (intact- and denuded-endothelium) precontracted with phenylephrine were incubated with T3 (10-8, 10-7, 10-6, 10-5, and 10-4 M), and tension was recorded using a force-displacement transducer coupled with an acquisition system. To assess the involvement of oxidative stress, aortic rings were preincubated with T3 and subsequently submitted to an in vitro reactive oxygen species (ROS) generation system. The level of significance adopted in the statistical analysis was 5%.

**Results:**

T3 (10-4 M) promoted vasorelaxation of phenylephrine precontracted aortic rings in both intact- and denuded-endothelium conditions. Aortic rings preincubated in the presence of T3 (10-4 M) also showed decreased vasoconstriction elicited by phenylephrine (1 µM) in intact-endothelium preparations. Moreover, T3 (10-4 M) vasorelaxation effect persisted in aortic rings preincubated with NG-nitro-L-arginine methylester (L-NAME, 10 µM), a nonspecific NO synthase (NOS) inhibitor. Finally, T3 (10-4 M) exhibited, in vitro, an antioxidant role by reducing NADPH oxidase activity and increasing SOD activity in the aorta’s homogenates.

**Conclusion:**

T3 exerts dependent- and independent-endothelium vasodilation effects, which may be related to its role in maintaining redox homeostasis.

## Introduction

In cardiac and cerebral diseases, important alterations in the vascular relaxation mechanisms occur, promoting a blood flow reduction frequently associated with an ischemic situation.^1^ Systemic arterial hypertension, myocardial infarction, pulmonary hypertension, and stroke are examples of vascular injury conditions related to vasocontraction and impairment of circulatory system.^2^ The exacerbated response to catecholamines, inflammatory mediators, and reactive oxygen species (ROS) exert a relevant role in the endothelial dysfunction.^3^ In this context, oxidative stress plays an important role since ROS induce vasoconstriction and adverse vascular remodeling.^4^ Besides that, increased vascular tonus produces an augmented resistance to the blood flow, generating shear-stress. This condition has critical effects on endothelial homeostasis, causing elevated ROS production. Several cellular sources, such as mitochondria, endoplasmic reticulum, and peroxisomes, can promote ROS formation. In addition, enzymes such as xanthine oxidase and Nicotinamide adenine dinucleotide phosphate oxidase (NADPH oxidase/Nox) also have the ability to synthesize these chemical species.^5^

NADPH oxidase is the main enzyme in the vessels that synthesizes superoxide anion radical (O_2_^•−^).^6^ O_2_^•−^ molecule can be converted to hydrogen peroxide (H_2_O_2_) in a reaction catalyzed by superoxide dismutase (SOD). From this perspective, H_2_O_2_ plays a dual role since not only can it produce vasodilation in cerebral, coronary, and pulmonary arteries,^7^ but it can also act as a relevant vasoconstrictor in peripheral arteries and in the aorta.^8^ Besides that, O_2_^•−^ can combine with nitric oxide (NO), resulting in peroxynitrite (ONOO^−^) formation.^9^ ONOO^−^ is a potent oxidant agent of biological membranes and can induce cell death. On this basis, finding therapeutic approaches that combine both vasodilatation and antioxidant actions is important to treat vascular diseases.^10^

Thyroid hormones (TH) have been shown to promote cardioprotective and vasoprotective effects in experimental models of heart diseases. In the infarction model, TH not only improved post-infarction adverse cardiac remodeling, but also presented an antioxidant action in cardiac and vascular tissue.^11,12^ Additionally, TH improved the expression of angiogenesis proteins, such as hypoxia-inducible factor 1α (HIF-1α) and vascular endothelial growth factor (VEGF).^12^ Moreover, T3 induces vasorelaxation through different mechanisms. In an experimental model of a salt overload diet, the vessels incubated with T3 increased their vasodilatation and decreased ROS production.^13^ NO levels elevation and protein kinase G (PKG)/vasodilator-stimulated phosphoprotein (VASP) and phosphoinositide-3-kinase (PI3K)/ protein kinase B (AKT) pathways activation appear to be the main processes through which TH promotes vasorelaxation.^14^ Nevertheless, T3 also exerts its vasodilatory function through an endothelium-independent manner, suggesting a direct action of TH on smooth muscle cells.^15^

Despite studies demonstrating a protective action of TH in the heart and vessels,^11,12^ it is still necessary to better elucidate the role of T3 on vascular tone and oxidative stress in vessels. Accordingly, it is necessary to further explore the T3 vasodilator mechanism and the impact of ROS on this process. For this purpose, the objective of this study was to evaluate T3 impact on vascular response in isolated vessels regarding the influence of endothelium and the involvement of oxidative stress parameters in this context.

## Methods

### Animals

Forty-five-day-old male Wistar rats (weighing 202 ± 25 g) (N = 60) from the Laboratory Animal Reproduction and Experimentation Center were used. The animals were allocated under standard vivarium conditions: controlled temperature environment (21 ± 2 °C), 12-hour light-dark cycle and 70% relative humidity; water and standard rodent chow were offered “ad libitum.” The animals were also acclimatized for seven days before the start of the experimental protocol. All procedures in this study were in accordance with the guidelines for Biomedical Research Involving Animals from the Council for International Organizations of Medical Science (CIOMS) and approved by the Ethics Committee on the Use of Animals (protocol number: 38964).

### Sample size calculation

The calculation of the sample size was performed using the G*Power 3.1.9.2 software (Schleswig-Holstein, Germany). Probability of error α = 0.05, statistical test power = 0.80 and effect size = 0.4 were considered.

### Vessel preparation for vascular reactivity

Rats were anesthetized with ketamine (90 mg/kg, intraperitoneal) and xylazine (10 mg/kg, intraperitoneal) simultaneously and euthanized by decapitation. After euthanasia, the aortas were quickly isolated and kept in Krebs-Henseleit buffer solution (NaCl 115 mM, CaCl_2_ 2.5 mM, KCl 4.6 mM, KH_2_PO_4_ 1.2 mM, MgSO_4_. 7 H_2_O 1.2 mM, NaHCO_3_ 25mM, dextrose 11.1 mM, Na_2_EDTA 3 mM), at 37 °C and pH 7,4, under 95% O_2_–5% CO_2_. Adipose and connective tissues were carefully removed, and the aortas were cut into cylindrical segments of 4 mm in length. In some experiments, the endothelium was mechanically removed by gently rubbing the inner surface of the aorta’s segments using a stainless-steel wire. Two hooks of stainless-steel wire bent in a modified L-shape were used to mount each ring in organ bath chambers (AVS Projetos, São Carlos, SP, Brazil). The short, straight portion of each hook passed through the lumen of the ring. The lower hook was attached to the base of the organ chamber filled with 10 mL of Krebs-Henseleit buffer solution, and the upper hook to a strain gauge. Isometric tension was recorded using a force-displacement transducer (TIM-100; AVS Projetos, São Carlos, SP, Brazil) connected to an acquisition system (AQCAD 2.0.5; AVS Projetos, São Carlos, SP, Brazil). The segments were subjected to a basal tension of 1.0 gf, which was adjusted every 15 minutes during an initial 45-minute equilibration period.

The integrity of the endothelium was verified through incubation with acetylcholine (10 µM). A relaxation equal to or greater than 90% of maximum contraction induced by phenylephrine (1 µM) was considered demonstrative of the functional integrity of the endothelium. Each ring was sequentially washed with Krebs-Henseleit buffer solution, re-equilibrated for 45 min and basal tensions were recorded.

### Vascular reactivity studies

The experimental incubation protocol is represented as follows: a) verification of a T3 dose-response curve (10^-8^ to 10^-4^ M) on both intact- and denuded-endothelium aortic segments precontracted with phenylephrine 1µM; b) pre-exposition of both intact- and denuded-endothelium vessels to T3 (10^-4^M), to show the lowest phenylephrine induced contractile response; c) verification of T3 (10^-4^M) on intact-endothelium aortic segments precontracted with phenylephrine 1µM and exposed to a nonspecific NO synthase (NOS) inhibitor, N^G^-nitro-L-arginine methylester (L-NAME; 10 µM) for 20 min. T3 was diluted in Krebs-Henseleit buffer, and solutions were prepared daily and protected from light. The control group consisted of the addition of the same volumes of Krebs-Henseleit buffer solution without T3.

### Preparation of aorta for in vitro assays

Aortas were homogenized for 40 seconds (OMNI Tissue Homogenizer, OMNI International, Kennesaw, GA, USA) in the presence of RIPA buffer 1:10 (5ml/g of tissue) with fluoride phenyl-methyl-sulfonyl (PMSF) (100 mM) 1% (v/v). The homogenate was centrifuged for 10 min at 8,000x*g* in a refrigerated centrifuge (4 °C) (Sorvall RC 5B – Rotor SM 24, Sorvall, Waltham, MA, USA), and the supernatant was collected for further analysis.

### Hydroxyl radical generation system

To verify the role of T3 on redox balance, the aorta’s homogenate supernatants were incubated with T3 (10^-4^M) for 15 min at 37°C. The group that did not receive T3 was incubated with distilled water in the same volume. After this, samples were incubated for 30 min at 37°C with FeCl_2_ (20µM), H_2_O_2_ (50mM), and ascorbic acid (1mM) to induce oxidative stress (hydroxyl radical generation system).^16^ These samples were used for biochemical evaluation.

### Biochemical analyses

#### Dosage of proteins

Proteins were quantified using the method described by Lowry et al.^17^ and were measured in a spectrophotometer (Anthos Zenyth 200 rt, Biochrom, Cambridge, UK) at 625 nm. The results were expressed in mg/mL.

#### Total ROS determination

Total ROS levels in the vessel homogenates were detected using 2′,7′-dichloro-dihydro-fluorescein diacetate (DCFH-DA), as previously described.^18^ DCFH-DA is oxidized to fluorescent 2,7-dichlorofluorescein (DCF) in the presence of ROS. Samples were excited at 488 nm, and the emissions were collected with a filter of 525 nm (LS 55 Fluorescence Spectrometer, Perkin Elmer, Waltham, MA, USA). Results were expressed in nmol DCF/mg protein.

#### Lipid peroxidation

Lipid peroxidation was evaluated by the production of chemiluminescence initiated by the addition of tert-butyl hydroxide (TBOOH).^19^ Chemiluminescence was measured in a scintillation spectrometer (LKB Wallac Rack Beta Liquid Scintillation Spectrometer 1215, Australia) operating as a luminometer and with a phototube emission sensor in the range of 380–620 nm. Results were expressed as counts per second cps/mg protein.

#### NADPH Oxidase Activity

NADPH oxidase activity was based on monitoring NADPH consumption at 340 nm (Anthos Zenyth 200 rt, Biochrom, Cambridge, UK). The results were expressed as nmol NADPH/min/mg protein.^20^

#### Superoxide Dismutase (SOD) activity

Determination of SOD activity was based on the inhibition of the reaction of the superoxide radical anion with pyrogallol.^21^ The results were expressed as U SOD/mg protein.

#### Catalase activity

Catalase activity was evaluated by measuring hydrogen peroxide decomposition rate spectrophotometrically at 240 nm.^22^ The results were expressed in nmol/min/mg protein.

## Statistical analysis

All data sets were submitted to Shapiro-Wilk normality test. Experiments testing different doses of T3 over time compared to Control were analyzed by factorial ANOVA complemented with Bonferroni’s post-hoc test for multiple comparisons. Experiments comparing fixed dose of T3 (10^-4^ M) pre-incubation effect on vasoconstriction elicited by phenylephrine and T3 (10^-4^ M) effect on aortic rings pre-incubated with L-NAME to Control group were analyzed by unpaired T-tests. Redox balance experiments were analyzed by two-way ANOVA complemented with Bonferroni’s post-hoc test for multiple comparisons apart from total ROS levels, which was analyzed using Kruskal-Walli’s test complemented with Dunn’s post-hoc test for multiple comparisons. Results were expressed as mean ± standard deviation for all data sets but total ROS levels, whose results were plotted as a scatter dot plot graphic depicting the median and the interquartile range for each data set. GraphPad Prism 8.0.2 for Windows (GraphPad Software, San Diego, CA, USA) was used to analyze data. Data were considered significant when p < 0.05.

## Results

### T3 promotes relaxation of aortic segments

Considering the intact vessel, T3 at 10^-4^M induced relaxation of phenylephrine-induced precontracted aortas compared to the control group (Figure 1A). Removal of endothelium did not abrogate the T3 (10^-4^M)-induced vasorelaxation effect (Figure 1B).

**Figure 1:** – Effect of T3 on vascular relaxation of aortic segments (A) in the presence of endothelium (END+) and (B) in the absence of endothelium (END-) precontracted with phenylephrine. Values represented as mean ± standard deviation (SD), n = 6-8 aortic rings per group. Factorial ANOVA followed by Bonferroni’s post-test. *Significant difference between Control and T3 (10-4 M) groups; p < 0.05.

### T3 decreases vasocontraction response induced by phenylephrine

Pre-exposition of intact aortic segments to T3 (10^-4^M) resulted in a reduced phenylephrine-induced contraction compared to the control group (Figure 2A). Nevertheless, the same outcome was not observed in preparations without the endothelium (Figure 2B).

**Figure 2:** – Effect of T3 (10-4 M) pre-incubation on vasocontraction of aortic segments elicited by phenylephrine (A) in the presence of endothelium (END+) and (B) in the absence of endothelium (END-). Values represented as mean ± standard deviation (SD), n = 6-10 per group. Unpaired t-test. *Significant difference between Control and T3 groups; p < 0.05. ∆T/aorta weight = ratio of tension variation by vessel weight.

### T3 exhibits a NOS-independent vasorelaxation effect

Intact-endothelium vessels were preincubated with L-NAME, an unspecific inhibitor of NOS, prior to T3 (10^-4^M) incubation. Even in the presence of L-NAME, T3 (10^-4^M) still resulted in relaxation (~20%) of phenylephrine-induced precontracted aortic segments as compared to the control group (Figure 3).

**Figure 3:** – Effect of T3 (10-4 M) on vascular relaxation of intact-endothelium aortic rings preincubated with L-NAME. Values represented as mean ± standard deviation (SD), n = 6 per group. Unpaired t-test. *Significant difference between Control and T3 groups; p < 0.05.

### T3 reduces NADPH oxidase activity and improves SOD activity *in vitro*

Even though ROS total concentration and lipid oxidation were not different between the groups (Figure 4A and 4B), NADPH oxidase activity was reduced when samples were exposed to T3 (10^-4^M) compared to control groups (Figure 4C). SOD activity was increased in T3 (10^-4^M) incubated vessels compared to control groups (Figure 4D). Catalase activity was increased only in the groups that received the oxidative stress induction (Figure 4E).

**Figure 4:** – Effect of T3 (10-4 M) incubation on (A) level of total reactive oxygen species (ROS), (B) lipid oxidation, (C) NADPH oxidase activity, (D) superoxide dismutase (SOD) activity and (E) catalase activity in aorta’s homogenates. Values represented as median with interquartile range for (A) and mean ± standard deviation (SD) for (B) to (E), n = 4-6 per group. Kruskal-Walli’s test was complemented with Dunn’s post-hoc test for (A), and two-way ANOVA was complemented with Bonferroni’s post-hoc test for (B) to (E). *Significant difference between Control and T3 groups in two-way ANOVA; p<0.05. #Significant difference between (OS-) and (OS+) groups; p<0.05. OS- = groups without oxidative stress (hydroxyl radical generation system) induction; OS+ = groups with oxidative stress (hydroxyl radical generation system) induction.

## Discussion

The main results of the present study comprise that T3 induced vasodilatation of intact- and denuded-endothelium aortic rings precontracted with phenylephrine in experimental model *ex vivo*, whose protocol was designed for vascular reactivity evaluation. Moreover, T3 plays a pivotal role in redox homeostasis, since this hormone reduces NADPH oxidase activity and increases SOD activity of aortic vessels *in vitro* (Central Illustration).

Oxidative stress, hypoxia, atherosclerosis, and inflammation are examples of triggers of vasoconstrictor events, which promote an imbalance between vasodilation and vasoconstriction, adverse vascular muscle remodeling and endothelial damage.^23^ Together, these factors support many diseases such as stroke, myocardial infarction, and hypertension.^24^ Therefore, it is important to explore the underlying mechanisms of vessel reactivity to propose therapeutic alternatives that may contribute to vascular disease management. Our experimental model focused on the evaluation of the vascular response of isolated aortas in the presence of T3. Incubation with phenylephrine induced contraction of aortic rings, which were subsequently subjected to different doses of T3. Our results showed that T3, at a concentration of 10^-4^ M, reduced phenylephrine-induced vasoconstriction. Corroborating with our data, Carrillo-Sepulveda et al.,^13^ using aortas from female Dahl salt-sensitive rats subjected to a high-salt diet, showed that the pre-incubation with T3 reduced the hypercontractile state of the vessels and improved the vasodilator response. In the experimental model of myocardial infarction, Ortiz et al.^12^ found that treatment with T3 and T4 (2 and 8 μg/100 g/day, respectively, orally for 12 days) augmented the immunocontent of VEGF and HIF-1α in the aorta of infarcted rats, which are proteins involved with angiogenesis in a hypoxic scenario. Additionally, VEGF also induces vasodilatation.^25^

Evaluating endothelium involvement in TH vasodilatation effect, our results showed that T3 administration caused a reduction in vascular constriction induced by phenylephrine in aortas with and without endothelium. However, in the aortas preincubated with 10^-4^ M T3, the absence of endothelium significantly affected its capability to prevent an increase of vascular tension evoked by phenylephrine. In view of that, it is possible that, in intact vessels, T3 can promote vasodilatation through a combination of endothelium-dependent and endothelium-independent mechanisms. In vessels without endothelium, vasodilation is maintained on account of independent mechanisms, but the ability of T3 to reduce vascular tension decreases. These results highlight the relevance of endothelium in the total effect of T3 on vessels. Due to this result, it was necessary to investigate the role of NO in the process of vasodilation induced by T3, since endothelium is an important source of this vasodilator molecule.

The role of T3 on the endothelium and the vasodilator mechanisms involved in this relationship have been explored in recent years, highlighting the relevance of NO-dependent signaling pathways.^26^ In addition, TH is capable of increasing the activity of nitric oxide synthase in erythrocytes, favoring the production of NO in the blood and improving NO bioavailability in the circulation.^27^ In the present study, when aortic rings were pre-incubated with L-NAME, a known NOS inhibitor, T3 vasodilatory action persisted. Such data support the hypothesis that T3 may also induce vasodilatation through a NO-independent and endothelium-independent mechanism. These results did not decrease the importance of the endothelium for the effect of this hormone; they demonstrated the capability of T3 to promote dilatation in the context where the endothelium is removed/damaged. Samuel et al.^28^ suggested that this NO-independent effect is associated with the modulation of the protein kinase G/vasodilator-stimulated phosphoprotein (PKG/VASP) pathway directly on the vascular smooth muscle cells. Furthermore, the antioxidant effect of low doses of thyroid hormones can also improve vascular dysfunction and contribute to a relaxed vascular tone.^12,13^

Although ROS are important in controlling vascular tone, when their concentrations exceed the tissue’s antioxidant capacity, oxidative stress is established. Disruption of redox homeostasis promotes vessel damage, oxidizes low-density lipoproteins, and causes atherosclerosis. The inflammatory process associated with these events leads to adverse vessel remodeling, decreasing vasodilator agents, and increasing the level of vasoconstriction.^29^ Our results show that incubation with T3 reduced NADPH activity in the aorta under experimental oxidative stress conditions established *in vitro*. In fact, NADPH oxidase plays an important role in the formation of ROS in blood vessels. These species can promote an oxidized cellular environment, leading to NOS uncoupling and increased production of superoxide anion radicals instead of NO. Furthermore, free radicals, such as superoxide anion, often act as NO scavengers, reducing their bioavailability and impairing vasodilatation.^30^ In this regard, Ortiz et al.^12^ also found a reduction in the activity of NADPH oxidase in the aorta of infarcted rats treated with thyroid hormones, while De Castro et al.^11^showed that TH administration is capable of reducing ROS in the heart of infarcted rats. In terms of total ROS levels, however, the present study did not find differences among groups. A similar result was found for lipid oxidation evaluation. These results may have arisen since aortas from healthy animals were used. Within this context, these vessel samples were induced to oxidative stress through the generation of a hydroxyl model, in which samples were incubated with FeCl_2_, H_2_O_2_ and ascorbic acid for 30 min at 37°C. In a previous study evaluating the aorta of infarcted rats, however, TH administration promoted a decrease in ROS levels.^12^ Nonetheless, the ability of T3 to decrease NADPH oxidase activity already demonstrates a vasoprotective effect of this hormone. Furthermore, when we evaluated antioxidant enzymes, T3 administration induced an increase in SOD activity in vascular tissue. SOD converts the radical superoxide anion into hydrogen peroxide, reducing the levels of this free radical, which supports a state of increased NO bioavailability, consequently improving the vessel’s relaxation capacity. In fact, our results show that T3 promotes a scenario that favors an improvement in NO bioavailability, contributing to the mechanism of endothelium-dependent vasodilation. These data corroborate other studies that show the vasoprotective role of thyroid hormones.^12^ When we evaluated catalase activity, only the aortas submitted to oxidative stress presented an increase in this enzyme activity. These results indicate a compensatory mechanism of the enzymatic antioxidant system in response to redox imbalance; however, T3 administration did not modify catalase activity. Concerning this result, a study evaluating the TH effects of erythrocytes of infarcted rats demonstrated an increase in catalase activity in the blood cells of treated animals.^27^ In view of this, it is possible that, under the effect of T3, erythrocytes will contribute more to the activity of this antioxidant enzyme than the vessel wall itself.

### Limitation of the study

The impossibility of exploring protein expression directly in the aortic rings used in vascular reactivity was a limitation of the study. Evaluating the expression of proteins such as endothelial nitric oxide synthase (eNOS) and signaling PIK3/AKT pathway, as well as antioxidant proteins, such as Nuclear factor erythroid 2-related factor 2 (NRF2), in the same sample incubated with T3 and subjected to vasoconstriction and vasodilation protocols, would have contributed to the mechanistic understanding of observed outcomes. However, the small size of the aortic rings and the wires used in the vascular reactivity protocol reduce the degree of reliability for subsequent molecular biology experiments using the same sample. Another limiting aspect of this article is the lack of evaluation of other resistance vessels, such as small arteries and arterioles, to check whether the effect of T3 on the aorta could be extrapolated. Indeed, the contractile state of small arteries and arterioles is responsible for defining vascular resistance, which is a key determinant of blood pressure. Structurally, resistance arteries have one or two layers of the vascular smooth muscle cell (VSMC), while conduit arteries, such as the aorta, have about 15 layers of VSMC. In this context, the endothelial cells of resistance arteries project the plasmatic membrane to the VSMC layer through internal elastic lamina (myoendothelial projections). The myoendothelial projections connect to the VSMC through gap junctions that facilitate electrical communication between the two cell layers.^31^ In this context, the hyperpolarization of endothelial cells is transmitted to the VSMC, consequently relaxing the vessels; therefore, this is the main mechanism for endothelium-dependent vasodilation of resistance arteries. On the other hand, such a mechanism differs for the aorta, for example, since myoendothelial projections are often absent in conduit vessels. Thus, NO-mediated vasodilatation is the primary mechanism in this scenario.

## Conclusion

Based on the data obtained, it is possible to conclude that T3 has an important vasodilator role, which is maintained even in the absence of the endothelium. In addition, this hormone can decrease the activity of the pro-oxidant enzyme NADPH and increase the activity of the antioxidant enzyme SOD. These results point to a beneficial effect of this hormone on vascular redox balance and peripheral resistance. This scenario favors a greater bioavailability of NO, which also contributes to the endothelium-dependent vasodilation mechanism. Within this context, these data endorse other studies that demonstrate the beneficial effects of thyroid hormones on the circulatory system.
